# Peptidomic analysis of follicular fluid in patients with polycystic ovarian syndrome

**DOI:** 10.3389/fcell.2023.1289063

**Published:** 2023-11-08

**Authors:** Ningyu Sun, Yuanyuan Chen, Lu Lu, Hua Yan, Jing Zhou, Kai Li, Wuwen Zhang, Lihua Yuan, Boon Chin Heng, Weiwei Zeng, Yin Shi, Guoqing Tong, Ping Yin

**Affiliations:** ^1^ Department of Reproductive Medicine, Shuguang Hospital Affiliated to Shanghai University of Traditional Chinese Medicine, Shanghai, China; ^2^ Department of Gynecology of Traditional Chinese Medicine, Changhai Hospital, Naval Medical University, Shanghai, China; ^3^ Shandong University of Traditional Chinese Medicine, Jinan, Shandong, China; ^4^ School of Stomatology, Peking University, Beijing, China; ^5^ Department of Gynecology, Shuguang Hospital Affiliated to Shanghai University of Traditional Chinese Medicine, Shanghai, China; ^6^ Key Laboratory of Acupuncture and Immunological Effects, Yueyang Hospital of Integrated Traditional Chinese and Western Medicine Affiliated to Shanghai University of Traditional Chinese Medicine, Shanghai, China; ^7^ Department of Reproductive Medicine, The First Affiliated Hospital of Xi’an Jiaotong University, Xi’an, Shaanxi, China

**Keywords:** peptidomics analysis, assisted reproduction, polycystic ovarian syndrome, follicular fluid, *in vitro* fertilization

## Abstract

**Objective:** The aim of this study was to analyze and compare the differential expression of peptides within the follicular fluid of polycystic ovary syndrome (PCOS) patients *versus* normal women by using peptidomics techniques. The underlying mechanisms involved in PCOS pathogenesis will be explored, together with screening and identification of potential functional peptides via bioinformatics analysis.

**Materials and methods:** A total of 12 patients who underwent *in vitro* fertilization and embryo transfer (IVF-ET) at the Reproductive Medicine Center of Shuguang Hospital Affiliated to Shanghai University of Traditional Chinese Medicine from 1 September 2022 to 1 November 2022 were included in this study. The follicular fluid of PCOS patients (*n* = 6) and normal women (*n* = 6) were collected. The presence and concentration differences of various peptides were detected by the LC-MS/MS method. GO and KEGG analysis were performed on the precursor proteins of the differentially-expressed peptides, and protein network interaction analysis was carried out to identify functionally-relevant peptides among the various peptides.

**Results:** A variety of peptides within the follicular fluid of PCOS *versus* normal patients were detected by peptidomics techniques. Altogether, 843 upregulated peptides and 236 downregulated peptides were detected (absolute fold change ≥2 and *p* < 0.05). Of these, 718 (718 = 488 + 230) peptides were only detected in the PCOS group, while 205 (205 = 174 + 31) were only detected in the control group. Gene Ontology enrichment and pathway analysis were performed to characterize peptides through their precursor proteins. We identified 18 peptides from 7 precursor proteins associated with PCOS, and 4 peptide sequences were located in the functional domains of their corresponding precursor proteins.

**Conclusion:** In this study, differences in the follicular development of PCOS *versus* normal patients were revealed from the polypeptidomics of follicular development, which thus provided new insights for future studies on the pathological mechanisms of PCOS development.

## 1 Introduction

Polycystic ovary syndrome (PCOS) is a common endocrine disease in women. Its clinical features include hyperandrogenism, ovulation disorders and polycystic changes within the ovary. Among women of childbearing age in China, the incidence of PCOS is 5.6% (Li et al., 2013), as compared to 7%–15% worldwide (Collée et al., 2021). Abnormal oocyte development and ovulation disorders due to PCOS are major causes of infertility in women.

Follicular fluid is the main nurturing environment for oocyte development, and the various components of follicular fluid is indicative of the secretory functions of oocytes, granulosa cells and follicular theca cell (Revelli et al., 2009; Da Broi et al., 2018). The main components of follicular fluid include various proteins, steroids and metabolites (Channing et al., 1980; Rodgers and Irving-Rodgers, 2010), with many studies having analyzed the various mRNA and microRNA species, oxidative stress biomarkers and proteomics of follicular fluid (Anahory et al., 2002; Lee et al., 2005; Twigt et al., 2012; Sang et al., 2013; Lewandowska et al., 2017; Butler et al., 2019; Gongadashetti et al., 2021). However, there are still very few studies that have focused on the peptidomics of follicular fluid in PCOS patients. Therefore, we conducted peptidomics analysis of the follicular fluid of PCOS and normal patients to compare and identify differentially-expressed peptides that play key roles in the pathological mechanisms of PCOS.

## 2 Materials and methods

### 2.1 Patients

A total of 588 patients with or without PCOS, who underwent *in vitro* fertilization and embryo transfer (IVF-ET) at the Reproductive Medicine Center of Shuguang Hospital Affiliated to Shanghai University of Traditional Chinese Medicine from 1 October 2018 to 1 November 2022 were included in this study to demonstrate differences in clinical features and embryo quality between non-PCOS and PCOS patients. Of these 588 people, 12 patients’ follicular fluid from 1 September 2022 to 1 November 2022 were randomly selected and were used for peptidomic analysis. Clinical ethics approval was obtained from the Medical Ethics Committee of Shuguang Hospital affiliated to Shanghai University of Traditional Chinese Medicine (Code: 2022-1148-85-01).

The inclusion criteria for the Control group were as follows:

a) Patients undergoing IVF/ICSI treatment due to male factor infertility;
b) Between 22 and 35 years old;
c) Regular menstrual cycle and normal ovulation;
d) Controlled ovulation hyperstimulation (COH) with antagonist protocol.

The inclusion criteria for the PCOS group were as follows:

a) Patients who meet the Rotterdam criteria (Rotterdam ESHRE/ASRM-Sponsored PCOS Consensus Workshop Group, 2004) for diagnosis of PCOS;
b) Between 22 and 35 years old;
c) COH with antagonist protocol.

Exclusion criteria:

Patients with other underlying disease conditions or endocrine disorders, such as hyperprolactinemia, Cushing’s syndrome, and androgen-secreting tumors.

### 2.2 Sample collection

Both groups were treated with antagonist protocol for COH. All patients were injected with gonadotrophin (Gn) from the second to 3rd day of their menstrual period. On the 6th day of Gn injection or when there was more than one dominant follicle with a diameter ≥14 mm, GnRh-A (Sizek, Merck) at 0.125–0.25 mg/d was added to the administered dose. The dosages of Gn or GnRH-A were adjusted according to the follicular condition, as monitored by transvaginal B-ultrasound and blood hormone levels. When at least two dominant follicles reached a diameter ≥18 mm, Gn administration was discontinued and hCG (Lizhu, Zhuhai) was injected during the night to induce ovulation. Oocytes and follicular fluid were retrieved by ultrasound-guided transvaginal follicular puncture 34–36 h after hCG injection. The follicular fluid of PCOS patients (*n* = 6) and normal women (*n* = 6) were collected. 1mL aliquots of the collected follicular fluid samples were ultrafiltered by 3 and 10 k Ultrafiltration Spin Columns (RT-UFC501096-5, Millipore). The peptide mixture was desalted by C18 ZipTip, followed by lyophilization with SpeedVac.

### 2.3 LC-MS/MS analysis

Analysis of follicular fluid peptides was carried out with an online nano-flow liquid chromatography in tandem with mass spectrometry, utilizing an EASY-nanoLC 1200 system (Thermo Fisher Scientific, MA, United States) connected to a Q Exactive mass spectrometer (Thermo Fisher Scientific, MA, United States). Acclaim PepMap C18 (75 μm × 25 cm) as equilibrated with solvent A (A: 0.1% formic acid in water) and solvent B (B: 0.1% formic acid in ACN). The peptides were eluted using the following gradient: 2%–6% for 3 min; 6%–20% for 39 min; 20%–35% for 5 min; 35%–100% for 1 min; maintained 100% for 12 min.The mass spectrometer was run under data dependent acquisition (DDA) mode, and automatically switched between MS and MS/MS mode. The survey of full scan MS spectra (m/z 200-1500) was acquired in the Orbitrap with 70,000 resolution.

### 2.4 Bioinformatics analysis

The Blast2GO version 5 software was used for functional annotation and GOATOOLS was used to perform GO enrichment analysis (Conesa et al., 2005; Klopfenste et al., 2018). Pathway analysis was processed by KOBAS (Xie et al., 2011). COG&KOG Analysis was based on the Phylogenetic classification of proteins encoded in complete genomes project (Tatusov et al., 1997). The search tool STRING was used to analyse protein-protein interactions (PPI). The Cytoscape software was used to analyse the STRING-based DEGs interaction network. The Cytoscape plug-in tool MCODE was used to analyse the PPI network module to obtain the DEGs network hotspot module. MCODE score >4 and the number of nodes >5 were selected. The Cytoscape plug-in tool cytoHubba was used to identify key targets and sub-networks of complex networks. The top 30 genes with high degrees and with MCODE score ≥10 were utilized as the cut-off criterion (Jian and Yang, 2020). The InterPro 64.0 software was used to process protein sequence analysis and classification. The UniProt Database and SMART online tools were used to determine if the significantly different peptide sequences were located in the conserved structural domains of their protein precursors. The Open Targets Platform database was used to investigate protein precursors associated with disease pathology.

### 2.5 Statistical analysis

One-way ANOVA or paired sample *t*-test were used to assess all experimental data, with *p* < 0.05 being considered statistically significant. The threshold for screening differentially-secreted peptides was a multiple of absolute value ≥2.

## 3 Results

### 3.1 Differences in IVF outcomes between PCOS *versus* normal patients

A total of 508 patients in the Control group and 80 patients in the PCOS group were included. There were no significant differences in age, infertility duration, BMI, FSH, E2 and P between the Control *versus* PCOS group (*p* > 0.05), whereas AMH and LH of the PCOS group was higher than that in the Control group, as shown in Table 1. There were no significant differences in fertilization rates, cleavage rates and embryo formation rates between the Control *versus* PCOS group (*p* > 0.05), but the MII oocyte rate, embryo formation rate and oocyte utilization rate of the PCOS group were significantly lower than that of the Control group (*p* < 0.05), while the number of retrieved oocytes displayed the opposite trend. The IVF outcomes are shown in Table 2.

**TABLE 1:** Comparison of basic clinical data.

Variables	Group		Z-value	*p*-value
	Control (*n* = 508)	PCOS (*n* = 80)		
Age (years)	31.00 (29.00,34.00)	31.00 (29.00,33.00)	−0.231	0.817
Infertility duration	3.00 (1.00,5.00)	3.00 (2.00,4.00)	−0.049	0.961
BMI (kg/m^2^)	22.50 (21.10,25.15)	22.45 (20.60,26.08)	−0.198	0.843
AMH (ng/mL)	2.59 (1.85,3.64)	6.39 (4.96,8.86)	−12.318	0.000*
FSH(IU/L)	5.67 (4.92,6.78)	5.60 (4.82,7.17)	−0.421	0.674
LH(IU/L)	3.09 (2.22,4.16)	4.02 (3.58,4.18)	−5.098	0.000*
E2 (pmol/L)	129.00 (92.00,179.00)	118.00 (89.00,162.00)	−1.217	0.223
P (nmol/L)	0.60 (0.40,0.94)	0.60 (0.50,1.08)	−0.837	0.403

*p < 0.05.

**TABLE 2:** Comparison of IVF outcomes.

Variables	Group		Z or X^2^ value	*p*-value
	Control (*n* = 508)	PCOS (*n* = 80)		
No. of retrieved oocytes	8.00 (4.00,12.00)	12.00 (6.00,19.00)	−4.714	0.000*
MII oocyte rate (%)	91.90% (3962/4311)	81.06% (839/1035)	107.154	0.000*
Fertilization rate (%)	81.20% (3217/3962)	78.78% (661/839)	2.594	0.107
Cleavage rate (%)	96.46% (3103/3217)	97.13% (642/661)	0.742	0.389
Embryo formation rate (%)	58.88% (1827/3103)	51.40% (330/642)	12.175	0.000*
Oocyte utilization rate (%)	42.38% (1827/4311)	31.88% (330/1035)	38.202	0.000*

*p < 0.05.

### 3.2 Identification of differentially expressed peptides

**FIGURE 1:**
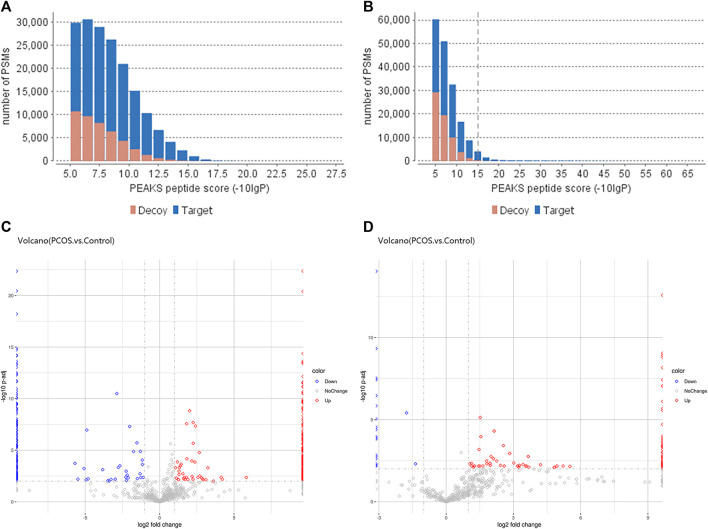
Differentially-expressed peptides within the follicular fluid of PCOS *versus* normal patients. The quality scores of 3 k peptide **(A)** and 10 k peptide **(B)** segments were correlated with the quantity distribution of corresponding spectra. The more blue bars with more than 20 scores, the higher the proportion of the results of high-quality spectra. **(C)** Volcano plot of differentially-expressed 3 k peptides detected in the follicular fluid of women with PCOS (*n* = 6) *versus* normal women without PCOS (*n* = 6). **(D)** Volcano plot of differentially-expressed 10 k peptides detected in the follicular fluid of women with PCOS (*n* = 6) *versus* normal women without PCOS (*n* = 6). The volcano plot is a scatter plot with the log2 value of Fold Change as the horizontal axis and the -log10 change value of *p*-value as the vertical axis. Based on the threshold of significant change as the dividing line, the data are mainly divided into three categories: red dot is upregulated, blue dot is downregulated, and gray dot with no change (refer to the legend). The distribution of different peptides within each group can be seen (this analysis is based on quantified total peptides).

We used PEAK software to measure peptide quality, as shown in Figures 1A, B. The peptides within the follicular fluid of the Control and PCOS groups were analyzed by Label-Free Quantification and LC-MS/MS. A total of 1354 10k peptides and 978 3k peptides were detected. Compared with the Control group, there were 471 differentially-expressed 3 k peptides (Fold Change≥2, *P* adj< 0.01), including 268 upregulated peptides and 203 downregulated peptides (Figure 1C), and 608 differentially-expressed 10 k peptides (Fold Change≥2, *P* adj< 0.01), including 575 upregulated peptides and 33 downregulated peptides (Figure 1D). A total of 230 3k peptides and 488 10k peptides were only detected in the PCOS group together with 174 3k peptides and 31 10k peptides were only detected in the Control group.

### 3.3 GO enrichment analysis

**FIGURE 2:**
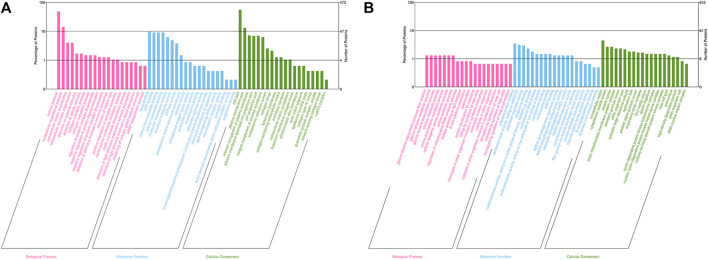
GO analysis of precursor proteins from peptides within the follicular fluid. **(A)** The top 20 categories of 3 k peptides with the smallest *p*-values in the GO classification chart. **(B)** The top 20 categories of 10 k peptides with the smallest *p*-values in the GO classification chart. The ordinate represents the number of proteins in each classification and its percentage of the total amount of differentially-expressed proteins, with the different colors representing different first-level classification, Biological Process (pink), Molecular Function (blue) and Cellular Component (green).

GO analysis was performed to determine the molecular functions (MF), cellular components (CC) and biological processes (BP) of the corresponding precursor proteins, and to predict the potential functions. In the Bar charts of GO classification, the top 20 pathways with the smallest *p* values were selected, as displayed in Figure 2. For the 3 k peptides, the top biological processes (BP) enriched by DEGs were mainly cellular processes, their molecular functions (MF) were mainly involved in drug binding, with the cellular component (CC) results showing that these genes were mainly involved in cell functions (Figure 2A). For the 10 k peptides, their BP were mainly involved in glycine betaine biosynthetic processes from choline, the MF enriched by DEGs were mainly involved in unfolded protein binding, while the CC results showed that these genes were mainly involved in extracellular matrix (Figure 2B).

### 3.4 KEGG pathway enrichment analysis

In total, 12 KEGG signaling pathways were enriched by DEGs of 10 k peptides, including Metabolic pathways, Spliceosome, Biosynthesis of amino acids, Epstein-Barr virus infection, Ribosome, RNA transport, Legionellosis, Glycolysis/Gluconeogenesis, Viral carcinogenesis, Complement and coagulation cascades, Apoptosis and Phagosome (Table 3). 17 KEGG signaling pathways were enriched by DEGs of 3 k peptides, including Metabolic pathways, Protein digestion and absorption, MAPK signaling pathway, Oxytocin signaling pathway, cGMP-PKG signaling pathway, Olfactory transduction, Huntington’s disease, Focal adhesion, PI3K-Akt signaling pathway, Pathways in cancer, ABC transporters, ECM-receptor interaction, Purine metabolism, Calcium signaling pathway, Transcriptional misregulation in cancer, Regulation of actin cytoskeleton and HTLV-I infection (Table 4). Interestingly, metabolic pathways are commonly correlated with PCOS.

**TABLE 3:** KEGG pathway enrichment analysis of DEGs of 10 k peptides.

Pathway description	PathwayID	Input number
Metabolic pathways	hsa01100	39
Spliceosome	hsa03040	18
Biosynthesis of amino acids	hsa01230	15
Epstein-Barr virus infection	hsa05169	15
Ribosome	hsa03010	14
RNA transport	hsa03013	14
Legionellosis	hsa05134	13
Glycolysis/Gluconeogenesis	hsa00010	13
Viral carcinogenesis	hsa05203	13
Complement and coagulation cascades	hsa04610	12
Apoptosis	hsa04210	12
Phagosome	hsa04145	12

**TABLE 4:** KEGG pathway enrichment analysis of DEGs of 3 k peptides.

Pathway description	PathwayID	Input number
Metabolic pathways	hsa01100	16
Protein digestion and absorption	hsa04974	8
MAPK signaling pathway	hsa04010	8
Oxytocin signaling pathway	hsa04921	6
cGMP-PKG signaling pathway	hsa04022	6
Olfactory transduction	hsa04740	6
Huntington’s disease	hsa05016	6
Focal adhesion	hsa04510	6
PI3K-Akt signaling pathway	hsa04151	6
Pathways in cancer	hsa05200	6
ABC transporters	hsa02010	5
ECM-receptor interaction	hsa04512	5
Purine metabolism	hsa00230	5
Calcium signaling pathway	hsa04020	5
Transcriptional misregulation in cancer	hsa05202	5
Regulation of actin cytoskeleton	hsa04810	5
HTLV-I infection	hsa05166	5

### 3.5 KOG analysis

**FIGURE 3:**
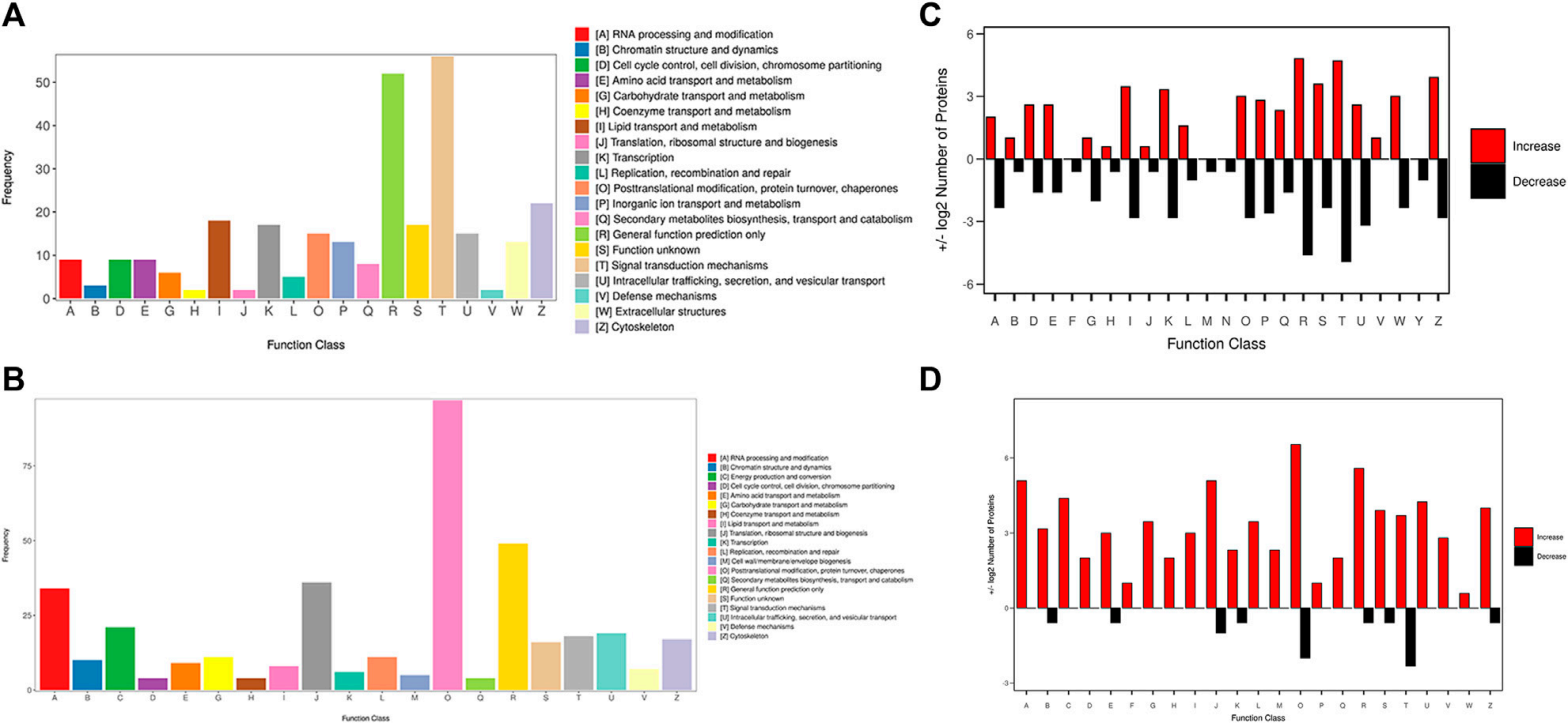
KOG function classification of the identified precursor proteins. **(A)** The function classification of the precursor proteins of 3 k peptides. **(B)** The function classification of the precursor proteins of 10 k peptides. Different colors in the bar charts indicate different KOG categories, and the *Y*-axis indicates the number of proteins belonging to each particular category. **(C)** Different KOG functional categories of upregulated/downregulated precursor proteins of 3 k peptides. **(D)** Different KOG functional categories of upregulated/downregulated precursor proteins of 10 k peptides. Upregulated proteins are depicted in red, downregulated proteins are depicted in black. The *X*-axis depicts various KOG categories, while the *Y*-axis represents the number of proteins that fall into these categories.

The KOG database was used to predict the function classification of the precursor proteins of different endogenous peptides based on sequence similarity. The bar chart shows the KOG function classification of the identified precursor proteins of 3 k peptides, which mainly involves post-translational modification, protein turnover, chaperones and general function prediction only (Figure 3A), the KOG function classification of the identified precursor proteins of 10 k peptides mainly involves Lipid transport and metabolism, general function prediction only, translation, ribosomal structure and biogenesis (Figure 3B). Different KOG functional categories of up-regulated/down-regulated precursor proteins are shown in Figures 3C, D.

### 3.6 PPI network construction and hub genes screening

**FIGURE 4:**
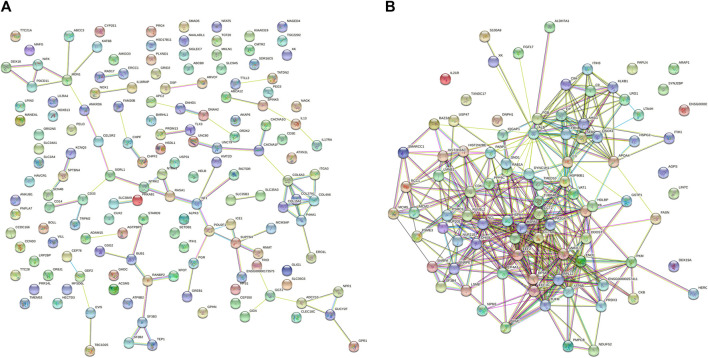
STRING network diagram. **(A)** PPI network diagram of 3 k peptides corresponding to precursor proteins. **(B)** PPI network diagram of 10 k peptides corresponding to precursor proteins.

The STRING database contains information about known and predicted protein interactions. The protein network diagram illustrates the whole network between proteins, with each node representing a protein. The names of each group of differentially-expressed proteins were submitted to the official website of STRING. After analysis, the protein interaction network diagram shown in Figure 4 can be obtained. The whole network of 3 k peptides has 360 nodes and 2493 edges (Figure 4A), while the whole network of 10 k peptides has 168 nodes and 1973 edges (Figure 4B).

**FIGURE 5:**
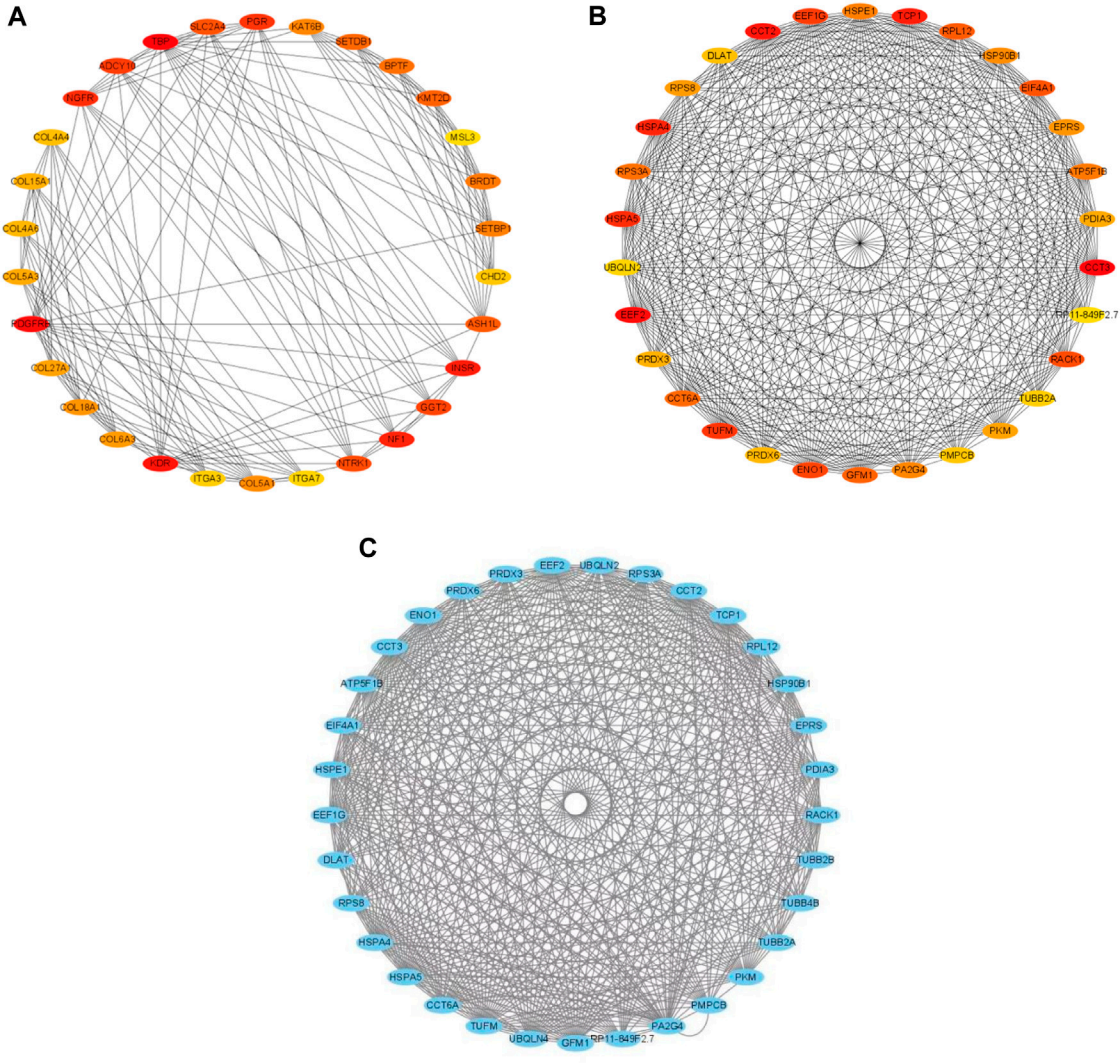
Identification of hub genes for PCOS. **(A)** The top 30 DEGs of 3 k peptides. **(B)** The top 30 DEGs of 10 k peptides. The top 30 DEGs in the interrelationship network analysed by degree in cytoHubba, the higher the rank, the redder the color. **(C)** The significant module identified from the PPI network using the molecular complex detection method (MCODE) with a score of >4.0 and nodes >5, MCODE score = 26.87.

The MCODE in the Cytoscape software were used to perform module analysis. In total, 4 modules of 3 k and 2 modules of 10 k with scores greater than 4 and nodes greater than 5 were detected. The top 30 genes with the highest degrees of connectivity (Figures 5A, B) and the genes with MCODE scores ≥10 were identified as hub genes. Finally, a total of 30 hub genes (All were derived from 10 k peptides) were screened (Figure 5C). KEGG analysis of these 30 genes revealed that they were mainly involved in Metabolic pathways and Ribosome. PCOS is a metabolic syndrome, which is consistent with hub genes mainly involved in metabolic pathways. Since no 3 k modules score is greater than 10 points, we selected modules with the highest score (9.860) and compared them with the genes of degree top 30, and identified three key genes (TBP, PDGFRB, INSR). We performed KEGG analysis on the top 30 3 k genes and found that they are primarily involved in protein digestion and absorption and the PI3K/Akt signaling pathway. The PI3K/Akt signaling pathway is necessary for insulin stimulation of glucose transport (Brozinick et al., 2003; Petersen and Shulman, 2018), and impaired PI3K/Akt signaling has been implicated in the development of IR (Etzion et al., 2010). In turn, IR would exacerbate the PI3K/Akt signaling pathway, forming a vicious cycle (Ren et al., 2021).

### 3.7 Identification and analysis of peptides associated with PCOS

The correlation of biologically active segmental peptides with precursor proteins has been previously reported (Dumont and Bischoff, 2010; Kawaguchi et al., 2010; Gao et al., 2014). We identified protein precursors of peptides associated with PCOS using a genome-wide open target platform (Jia et al., 2018). We found that 60 precursor proteins corresponding to 124 peptides were associated with PCOS. Then we analyzed the correlation between 30 Hub genes and PCOS and identified 7 precursor proteins corresponding to 18 peptides. The correlation between Hub genes and PCOS is shown in Table 5.

**TABLE 5:** Correlation between Hub genes and PCOS.

Gene name	Peptide sequences	Molecular weight	Theoretical pI	The estimated half-life	The instability index (II)	Aliphatic index	Fragment	Location	FDR	Association score with PCOS
HSPA5	KSDIDEIVLVGGSTRI	1701.94	4.56	1.3 h	18.51	133.75	420-435	-	0.007	0.0064
	RLTPEEIERM	1273.47	4.79	1 h	122.18	78	599-608	-	0.006	
HSPE1	KFLPLFDRV	1134.39	8.75	1.3 h	35.62	118.89	8-16	Cpn10	0.173	0.0146
HSP90B1	RLISLTDENALSGNEELTVKI	2315.61	4.41	1 h	15.59	130	116-136	low complexity	0.006	0.0421
	RTDDEVVQREEEAIQLDGLNASQIRE	3014.21	4.12	1 h	42.1	90	43-68	-	0.006	
PKM	REAEAAIYHLQLFEELRRL	2357.7	5.57	1 h	90.57	118.42	3-21	No domains	0.04	0.0092
	RRFDEILEASDGIMVARG	2035.31	4.78	1 h	60.09	92.22	113-130	No domains	0.05	
	REAEAAIYHLQLFEELRR	2244.54	5.57	1 h	95.04	103.33	3-20	No domains	0.056	
	RRFDEILEASDGIMVARGD	2150.39	4.44	1 h	57.45	87.37	113-131	No domains	0.007	
	RFDEILEASDGIMVARG	1879.12	4.32	1 h	29.34	97.65	114-130	No domains	0.049	
	KFGVEQDVDMVFASFIRK	2116.46	6.12	1.3 h	22.49	75.56	65-82	No domains	0.05	
PRDX6	RFHDFLGDSWGILFSHPRD	2302.54	5.99	1 h	10.3	61.58	34-42	No domains	0.006	0.0022
PRDX3	RDYGVLLEGSGLALRG	1675.91	6.07	1 h	10.8	121.88	170-185	No domains	0.007	0.0079
EEF2	KDGAGFLINLIDSPGHVDFSSEVTAALRV	3029.4	4.66	1.3 h	37.73	107.59	93-121	-	0	0.0421
	KAYLPVNESFGFTADLRS	2015.25	6.07	1.3 h	71.94	70.56	783-800	EFG_C	0.054	
	RVFSGLVSTGLKV	1362.64	11	1 h	−10.36	126.92	413-425	-	0.014	
	NLIDSPGHVDFSSEVTAALRV	2227.46	4.54	1.4 h	52.34	106.67	99-119	-	0.1	
	RALLELQLEPEELYQTFQRI	4699.3	4.49	1.4 h	59.54	114.15	160-179	EFG_IV	0.006	

## 4 Discussion

PCOS is a common reproductive endocrine disorder associated with metabolic diseases such as obesity, insulin resistance and type 2 diabetes (Barthelmess and Naz, 2014). In IVF treatment, although the number of oocytes obtained by PCOS patients are typically higher than that of non-PCOS patients, the oocyte maturation rate and oocyte quality are usually inferior, which often lead to lower fertilization rates, cleavage rates and embryo implantation rates in PCOS patients, as well as higher abortion rates (Qiao and Feng, 2011).

Currently, oocyte quality assessment is mainly based on morphological observations of the cumulus-oocyte complex, which is highly subjective and prone to human error. Follicular fluid is readily available during IVF treatment, and non-invasive metabolomics detection techniques can identify potential biomarkers and metabolic pathways associated with specific disease phenotypes of PCOS. It can be used as an indicator to predict oocyte quality and to supplement the evaluation of embryo morphology (Johnson et al., 2016). Follicular fluid constitute the direct growth environment of oocytes, consisting of various secretions produced by granulosa cells and serum for local capillary diffusion, as well as various plasma components that cross the blood follicular barrier, together with bioactive products secreted by granulosa cells and follicular theca cells (McRae et al., 2012; Basuino et al., 2016), which all play crucial roles in oocyte growth and development. Oocyte maturation disorder in PCOS patients may be related to changes in follicular fluid microenvironment caused by abnormalities in cytokine expression (Tumu et al., 2013; Niu et al., 2014). Because the developmental potential of oocytes directly determines the quality of embryos (Johnson et al., 2016), research on the microenvironment of oocyte growth and development together with in-depth analysis of the microenvironment of follicular fluid are helpful in finding a new method to evaluate the developmental potential of embryos and improve the outcome of ART. Therefore, rigorous in-depth investigations and analysis of the follicular fluid microenvironment is helpful in developing a new method to evaluate embryonic development potential and improve ART outcomes.

The metabolomics and proteomics profiles of follicular fluid in PCOS patients have been previously reported (Shen et al., 2022; Wang et al., 2022), but there are limited studies on follicular fluid peptidomics. To our knowledge, this study is the first to report the differences between PCOS *versus* normal patients in terms of follicular fluid peptidomics. Peptides are produced by hydrolysis of precursor proteins by endogenous peptidase. Physiological or pathological changes of the human body can be dynamically reflected in the production and metabolism of proteins and peptides (Schrader and Schulz-Knappe, 2001; Diamandis, 2006Diamandis, 2006). Many peptides play key roles in PCOS, such as GLP-1, LEAP-2 and ghrelin, Asprosin and XP-1 (Temur et al., 2017; Alan et al., 2019; Aslanipour et al., 2020; Cena et al., 2020), among which the GLP-1 receptor agonists have been proven to significantly reduce BMI and serum testosterone levels in PCOS patients (Niafar et al., 2016). Therefore, the analysis of follicular fluid polypeptidomics is of great significance for PCOS patients.

In this study, follicular fluid samples from patients with polycystic ovary syndrome and normal patients were collected for desalting using a C18 desalting column, and eluted peptide fractions were drained with a vacuum concentrator and then analyzed by mass spectrometry. To avoid the detection of peptides resulting from proteasomal degradation, protease inhibitors were added immediately after follicular fluid acquisition to reduce protein and peptide degradation. Altogether, 608 10 k differentially-expressed peptides and 471 3 k differentially-expressed peptides were detected in the six samples. We then performed bioinformatics analysis to characterize the functions and mechanisms of the differentially-expressed peptides. GO analysis showed that the differentially-expressed peptides were closely related to the regulation of mitochondrial RNA catabolic processes among various biological processes. PCOS patients are often characterized by poor oocyte quality, low fertilization rate and low embryo formation rate, and the ability of mitochondria to balance ATP supply and demand is considered to be the most critical factor for oocyte fertilization capacity and embryonic development potential. Damage to mitochondrial DNA, interruption of mitochondrial gene expression and decrease of mitochondrial membrane potential may lead to mitochondrial dysfunction in oocytes (Van Blerkom, 2011). KOG analysis showed that the precursor proteins corresponding to differentially-expressed peptides were mainly involved in post-translational modification, protein conversion, partner proteins, lipid transport and metabolism. As the main source of energy supply for the human body, the metabolism of sugars and lipids play a key role in biological processes. Disorders of glucose and lipid metabolism will lead to high blood fat and sugar levels, which will increase the amount of fat in PCOS patients and aggravate the inflammatory response. When glucose is transported to fat cells, excessive reactive oxygen species will be produced, which will increase the oxidative stress response. The combined effects of oxidative stress response and inflammatory response will cause cardiovascular damage and trigger an inflammatory cascade, with increased inflammation further accentuating oxidative stress (Uçkan et al., 2022). In order to identify key peptides implicated in PCOS pathophysiology, the Cytoscape software was used to visualize and analyze key genes, and 33 key genes were identified, including 30 10 k peptide genes and 3 3 k peptide genes. The biological functions of segmental functional peptides is related to the biological functions of their precursor proteins (Kawaguchi et al., 2010; Gao et al., 2014). Therefore, we identified protein precursors of differentially-expressed peptides associated with PCOS using a genome-wide open target platform (Wu et al., 2017). The results showed that 7 precursor proteins corresponding to 18 peptides were associated with PCOS. The expression levels of 18 peptides within the follicular fluid of PCOS patients were upregulated. The corresponding genes of the 18 peptides were HSPA5, HSPE1, HSP90B1, PKM, PRDX6, PRDX3 and EEF2. HSP5A, HSPE1 and HSP90B1 are proteins of the heat shock protein family, mainly involved in protein synthesis, transportation, folding and quality control (You et al., 2012), with all these functions being related to the pathological characteristics of PCOS. PKM catalyzes the final rate-limiting step of glycolysis by mediating the transfer of phosphate groups from phosphoenolpyruvate (PEP) to ADP, thereby producing ATP (Ashizawa et al., 1991; Dombrauckas et al., 2005; Vander Heiden et al., 2010). PRDX6 and PRDX3 belong to the PRDX family, which can catalyze the thiol-specific peroxidation of hydrogen peroxide and organic hydroperoxide for reduction to water and alcohol respectively, thereby playing a key role in cellular anti-oxidative stress protection by detoxifying peroxide (Tsuji et al., 1995; Cao et al., 2007; Yewdall et al., 2018; Rebelo et al., 2021). EEF2 catalyzes the GTP-dependent ribosome translocation step during translation extension, as well as catalyzing the coordinated movement of two tRNA molecules, mRNA, and ribosomal conformational changes. In addition, KEGG pathway analysis of DEGs showed that the metabolic pathways were enriched in 3 and 10 k peptides. PCOS patients are usually characterized by metabolic abnormalities, which is consistent with our results.

Subsequently, the SMART and SABLE softwares were used to analyze the positions of 18 peptides within the precursor proteins, of which 4 peptide sequences were located within the conserved domain. They were “KFLPLFDRV” (precursor protein HSPE1), “RLISLTDENALSGNEELTVKI” (precursor protein HSP90B1), “KAYLPVNESFGFTADLRS” and “RALLELQLEPEELYQTFQRI” (precursor protein EEF2). We will further study the role of these peptides in PCOS and the related underlying mechanisms involved.

In conclusion, we have demonstrated abnormal secretion of polypeptides within the follicular fluid of patients with PCOS. Our data thus provides a comprehensive analysis that enables a better understanding of the various functions and mechanistic effects of follicular fluid-derived secreted peptides.

## Data Availability

The mass spectrometry proteomics data which support the findings in the study are deposited to the ProteomeXchange Consortium via the PRIDE partner repository with the dataset identifier PXD045753. The data are openly available when the corresponding manuscript is published.
